# Greeting

**Published:** 1892-12

**Authors:** 


### EDITORIAL DEPARTMENT.

F. E. DANIEL, M. D., Editor.
A. J. SMITH. M. D., Galveston, Associate Editor.

### GREETING.

CHRISTMAS is here again! What a world of memories the word conjures up! What a variety of pictures it presents to the human mind. What a difference there is in its signification according to how we take it! To the Christian the word is sacred; it is full of hallowed memories; it presents to the mind the meek and lowly Nazarene laid in the humble manger in the far off Orient two thousand years ago. Ulike "the birds of the air, which have their nests" and "the foxes their holes," the "Son of Man had not where to lay his head." The Star arisen in the East—the wise men journeyed to behold him. That lowly Infant was GOD MADE MAN, the *Redeemer of the World!* What more natural, therefore, than that the anniversary of this advent upon earth should be celebrated by all His followers and made ever new and holy, and to live in the human heart forever, celebrated with joy and thanksgiving, with gratitude and prayer. It is a day for rejoicing in all Christian countries; but how differently is it "celebrated!" By some it is made the occasion of riotous mirth, of drinking and carousing; by some by rest from labor and innocent recreation; by others by devout prayer and gratitude. But with all, it is a time for rejoicing—it has become a "holiday" in the modern sense, and not, with all, a "holy-day." We congratulate each other on the return of the day, and we commemorate it and renew friendships in various ways and by exchanging tokens of remembrance.

To its many readers, and especially to its steadfast friends,—friends who saw its birth, witnessed and helped its growth, and now rejoice with us in its strong manhood, the JOURNAL extends greeting! It wishes them all continued health, happiness and prosperity; and in its humble way it contributes herewith to the Christmas enjoyment of its readers.

Wishing you all a Merry Christmas and a very happy New Year, the JOURNAL greets you!

